# Genotype-phenotype correlations for pancreatic cancer risk in Dutch melanoma families with pathogenic *CDKN2A* variants

**DOI:** 10.1136/jmedgenet-2019-106562

**Published:** 2020-06-01

**Authors:** Kasper A Overbeek, Mar DM Rodríguez-Girondo, Anja Wagner, Nienke van der Stoep, Peter C van den Akker, Jan C Oosterwijk, Theo A van Os, Lizet E van der Kolk, Hans F A Vasen, Frederik J Hes, Djuna L Cahen, Marco J Bruno, Thomas P Potjer

**Affiliations:** 1 Department of Gastroenterology & Hepatology, Erasmus University Medical Center, Rotterdam, The Netherlands; 2 Department of Biomedical Data Sciences, Leiden University Medical Center, Leiden, The Netherlands; 3 Department of Clinical Genetics, Erasmus University Medical Center, Erasmus MC Cancer Institute, Rotterdam, The Netherlands; 4 Department of Clinical Genetics, Leiden University Medical Center, Leiden, The Netherlands; 5 Department of Genetics, University Medical Center Groningen, University of Groningen, Groningen, The Netherlands; 6 Family Cancer Clinic, The Netherlands Cancer Institute, Amsterdam, The Netherlands; 7 Department of Gastroenterology & Hepatology, Leiden University Medical Center, Leiden, The Netherlands

**Keywords:** clinical genetics, gastroenterology, genetic screening/counselling, pancreas and biliary tract, oncology

## Abstract

**Background:**

Pathogenic variants in the *CDKN2A* gene are generally associated with the development of melanoma and pancreatic ductal adenocarcinoma (PDAC), but specific genotype-phenotype correlations might exist and the extent of PDAC risk is not well established for many variants.

**Methods:**

Using the Dutch national familial melanoma database, we identified all families with a pathogenic *CDKN2A* variant and investigated the occurrence of PDAC within these families. We also estimated the standardised incidence ratio and lifetime PDAC risk for carriers of a highly prevalent variant in these families.

**Results:**

We identified 172 families in which 649 individuals carried 15 different pathogenic variants. The most prevalent variant was the founder mutation c.225_243del (p16-*Leiden*, 484 proven carriers). Second most prevalent was c.67G>C (55 proven carriers). PDAC developed in 95 of 163 families (58%, including 373 of 629 proven carriers) harbouring a variant with an effect on the p16INK4a protein, whereas PDAC did not occur in the 9 families (20 proven carriers) with a variant affecting only p14ARF. In the c.67G>C families, PDAC occurred in 12 of the 251 (5%) persons at risk. The standardised incidence ratio was 19.1 (95% CI 8.3 to 33.6) and the cumulative PDAC incidence at age 75 years (lifetime risk) was 19% (95% CI 7.5% to 30.1%).

**Conclusions:**

Our results support the notion that pathogenic *CDKN2A* variants affecting the p16INK4a protein, including c.67G>C, are associated with increased PDAC risk and carriers of such variants should be offered pancreatic cancer surveillance. There is no clinical evidence that impairment of only the p14ARF protein leads to an increased PDAC risk.

## Introduction

The *CDKN2A* gene (MIM #600160) is a tumour suppressor gene encoding two distinct proteins using different first exons that are translated through alternative reading frames. The p16INK4a protein is coded by exons 1α, 2 and 3, while the p14ARF protein is coded by 1β and a part of exon 2.^1^ Thus, a germline mutation (henceforth referred to as *pathogenic variant*) can affect one or both of these proteins, depending on its location in the *CDKN2A* gene. Although both proteins act as tumour suppressors, they do so through different pathways. The p16-retinoblastoma pathway controls cell cycle G1 phase exit, whereas the p14ARF-p53 pathway induces cell cycle arrest or apoptosis.^2^ These properties suggest that *CDKN2A* variants may display specific genotype-phenotype correlations with risk of malignancy.

The most important malignancy associated with pathogenic variants in the *CDKN2A* gene is cutaneous melanoma, for which carriers have a lifetime risk of 70%.^3^ Pathogenic variants are found in up to 40% of high-density melanoma families, and *CDKN2A* is therefore the major high-risk susceptibility gene for hereditary cutaneous melanoma.^1 4^ The second most frequent tumour is pancreatic ductal adenocarcinoma (PDAC),^5–9^ for which the lifetime risk is up to 20%.^9–12^ This level of risk is among the highest for the known PDAC susceptibility genes,^12 13^ and pathogenic variants in the *CDKN2A* gene are even found in familial pancreatic cancer families that do not have cases of melanoma.^14–16^ Due to this high PDAC risk, carriers are eligible to be included in a pancreatic cancer surveillance programme.^17^

In melanoma families, pathogenic variants in the *CDKN2A* gene are found across the entire coding region and therefore may affect either p16INK4a, p14ARF or both.^3 6 18^ However, for the risk of PDAC, genotype-phenotype correlations for specific *CDKN2A* variants have been proposed, based on the observation that PDAC is only found in relation to variants affecting p16INK4A (with or without an effect on p14ARF).^6 7 19^ PDAC-associated variants include the founder mutation c.225_243del in exon 2, also known as p16-*Leiden*, the most commonly found pathogenic variant in the Netherlands,^20 21^ which conveys an estimated lifetime risk for PDAC of 15%–20%.^8 10^ Genetic testing of Dutch melanoma families also regularly identifies pathogenic variants other than p16-*Leiden,* 22 for which the exact PDAC risk is often uncertain.^19^ The uncertainty surrounding PDAC risk in non-p16-*Leiden* carriers precludes offers of surveillance within a pancreatic cancer surveillance research programme, as these programmes are reserved for individuals with a proven increased lifetime risk.^17^ The aim of this study was to further explore the possible genotype-phenotype correlation between pathogenic *CDKN2A* variants and PDAC risk. To this end, we assessed the occurrence of PDAC in Dutch melanoma families with a (likely) pathogenic *CDKN2A* variant. In addition, we determined the lifetime PDAC risk for the second most prevalent pathogenic variant in the Netherlands using detailed pedigree information.

## Methods

### Study design and data collection

Data for this study were derived from the previously described Dutch national database for familial melanoma.^22^ In this database, clinical information is stored on all melanoma families that have been tested for *CDKN2A* pathogenic variants at the Laboratory for Diagnostic Genome Analysis of the department of Clinical Genetics at Leiden University Medical Centre. This laboratory has been the primary sequencing facility for *CDKN2A* in the Netherlands since 1998, and performs sequencing for all clinical genetics departments in the Netherlands. According to Dutch referral guidelines, *CDKN2A* sequencing is indicated if the family meets one of the following criteria: (1) Two first-degree relatives with melanoma. (2) Two first-degree or second-degree relatives with melanoma and one first-degree or second-degree relative with PDAC. (3) Three or more primary melanomas in one individual. (4) An individual with melanoma under 18 years of age. (5) An individual with a history of both melanoma and PDAC.

Using this national database, we selected all melanoma families in which a pathogenic or likely pathogenic germline *CDKN2A* variant (class 4 or 5 variant)^23^ was identified during the period 1998–2015, regardless of the occurrence of PDAC in the family. Classification of these variants was based on previously reported co-segregation with disease and/or strong evidence of impaired protein function. Families with a variant of uncertain significance (class 3)^23^ were also included when the variant was shown through linkage analysis to be located on a pathogenic *CDKN2A* haplotype. The linkage criteria are based on haplotype mapping analysis data using eight simple polymorph tandem repeat microsatellite markers (region D9S1878–D9S162) encompassing the *CDKN2A* genomic region and neighbouring genes (9p13.3–9p22.1). Previous studies have shown that it is reliable to use simple tandem repeat polymorphism markers in this region to identify a common haplotype in different families.^24 25^ When variant carriers from a single large family (>8 meioses) or from different families carried a common haplotype for these markers per investigated variant (and healthy control family members and other control samples did not), the haplotype was classified as pathogenic.

Pedigrees were reviewed and updated for the occurrence of PDAC in any family member up to August 2018. Since the positive predictive value of self-reported family history for PDAC has been reported to exceed 75%,^26^ we included information on all reported PDAC diagnoses. For each individual family member, mutation status, history of any type of cancer, age at cancer diagnosis, age at last follow-up, and age and cause of death (if applicable) were updated by the referring clinical genetics department.

### Statistical analysis

Descriptive statistics were used to describe the prevalence of the different pathogenic *CDKN2A* variants and of PDAC within these families. For the second most prevalent variant (c.67G>C, p.(Gly23Arg)) we assessed the cumulative lifetime PDAC risk using detailed pedigree information. First, we excluded PDAC cases that were the index patient of their family, to prevent overestimation of risk as a result of ascertainment bias. We also excluded proven non-carriers, and all untested second-degree or higher-degree family members of a proven carrier, to prevent the effect of a likely reporting bias. Included in the analysis were proven carriers, obligate carriers, and untested first-degree family members of a proven or obligate carrier. Second, a standardised incidence ratio (SIR) was calculated, as the ratio of observed to expected cancers. The expected number of cancers was calculated based on the sum of all individual cumulative hazards derived from sex and age group-specific incidence rates for PDAC in the general Dutch population.^27^ Third, we estimated cumulative lifetime risk, defined as cumulative PDAC incidence by 75 years of age, using the Kaplan-Meier method. Mutation probabilities based on kinship coefficients were used as analytical weights in the Kaplan-Meier analysis, to avoid possible testing bias and increase efficiency. We calculated 95% CIs for both the SIR and the lifetime risk using bootstrapping at the family level (1000 repetitions).

## Results

### Pathogenic *CDKN2A* variants

**Figure 1:**
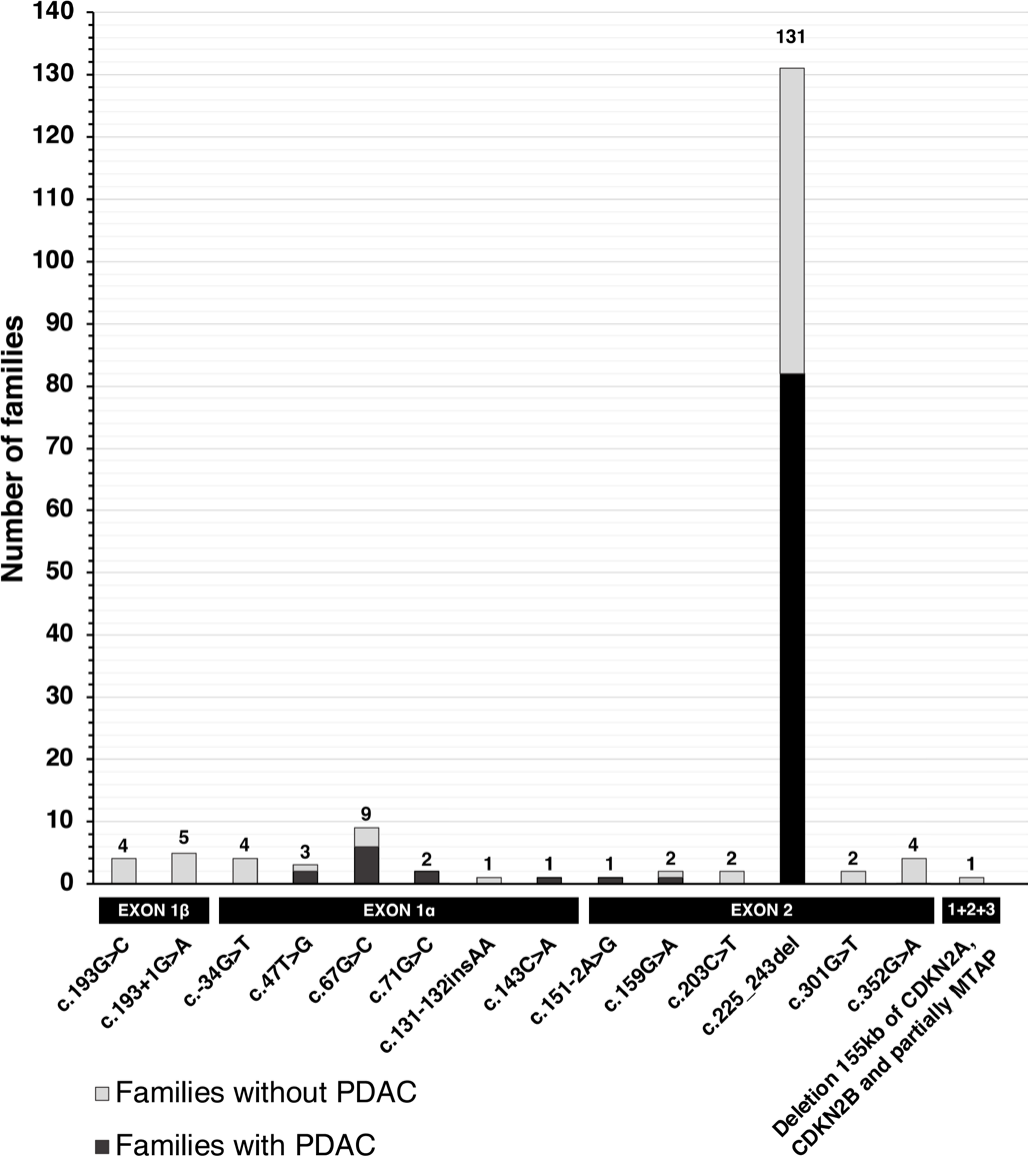
Visualisation of variant frequencies and pancreatic cancer occurrence. PDAC, pancreatic ductal adenocarcinoma.

Between 1998 and 2015, a total of 15 different (likely) pathogenic *CDKN2A* variants was found in 172 *CDKN2A*-mutated melanoma families that included a total of 649 proven variant carriers (table 1). Seven variants affected both the p16INK4a and p14ARF proteins (143 families, 543 proven carriers), six variants solely the p16INK4a protein (20 families, 86 proven carriers), and two variants solely the p14ARF protein (9 families, 20 proven carriers). The p16-*Leiden* variant (c.225_243del, p.(Ala76Cysfs*64)) was found in the majority of families (131/172 families, 76%). The frequencies of the 15 variants are presented in figure 1.

**Table 1:** Overview of pathogenic variants and pancreatic cancer occurrence

Variant location	*CDKN2A* nucleotide change*	*CDKN2A*/p16INK4a amino acid change	*CDKN2A*/p14ARF amino acid change	Total no. of families (no. of proven carriers)	No. of families with PDAC (no. of proven carriers in these families)	No. of PDAC cases (no. of validated† cases)	No. of proven carriers with PDAC + no. of obligate carriers with PDAC	PDAC occurrence in literature
Exon 1β	c.193G>C	None	p.(Gly65Arg)	4 (8)	0 (−)	0 (−)	0+0	No
Exon 1β	c.193+1G>A	None	p.? (splicing)	5 (12)	0 (−)	0 (−)	0+0	No
Exon 1α	c.-34G>T	p.?	None	4 (8)	0 (−)	0 (−)	0+0	Yes^6 7 15 19 37^
Exon 1α	c.47T>G	p.(Leu16Arg)	None	3 (9)	2 (5)	2 (0)	0+1	Yes^15 19^
Exon 1α	c.67G>C	p.(Gly23Arg)	None	9 (55)	6 (39)	12 (5)	5+4	No
Exon 1α	c.71G>C	p.(Arg24Pro)	None	2 (3)	2 (3)	2 (2)	2+0	Yes^6 7 15 19 37^
Exon 1α	c.131-132insAA	p.(Tyr44*)	None	1 (3)	0 (−)	0 (−)	0+0	Yes^15 19^
Exon 1α	c.143C>A	p.(Pro48Gln)	None	1 (8)	1 (8)	3 (1)	1+1	Variant not reported
Exon 2	c.151-2A>G	p.? (splicing)	p.? (splicing)	1 (12)	1 (12)	1 (0)	0+0	Variant not reported
Exon 2	c.159G>A	p.(Met53Ile)	p.(Asp68Asn)	2 (5)	1 (4)	1 (1)	0+0	Yes^15 19^
Exon 2	c.203C>T	p.(Ala68Val)	p.(Arg82Arg)	2 (7)	0 (−)	0 (−)	0+0	Yes^36^
Exon 2	c.225_243del	p.(Ala76Cysfs*64)	p.(Arg90Valfs*76)	131 (484)	82 (302)	NA	NA	Yes^6–8 10 15 16 19 37^
Exon 2	c.301G>T	p.(Gly101Trp)	p.(Arg115Leu)	2 (2)	0	0 (−)	0+0	Yes^6 7 14 15 19 37^
Exon 2	c.352G>A	p.(Ala118Thr)	p.(Gly132Asp)	4 (31)	0	0 (−)	0+0	Variant not reported
Exon 1+2+3	Deletion 155 kb of *CDKN2A*, *CDKN2B* and partially *MTAP*	p.? (whole gene deletion)	p.? (whole gene deletion)	1 (2)	0	0 (−)	0+0	Variant not reported

The variants c.143C >A and c.203C >T are located on a pathogenic haplotype.

*RefSeq NM_000077.4 isoform p16INK4a, RefSeq NM_058195.3 isoform p14ARF (for exon 1β).

†Through medical records and/or pathology reports.

*CDKN2A*, cyclin-dependent kinase inhibitor 2A; NA, not analysed; PDAC, pancreatic ductal adenocarcinoma.

### Pancreatic cancer occurrence

PDAC occurred in 95 families (55%, 373 proven carriers, table 1 and figure 1). Regarding variants affecting both proteins, 59% of the families with such a variant included cases of PDAC and these families accounted for 59% of the proven carriers of these variants. For variants affecting solely p16INK4a, PDAC was observed in 55% of the families, accounting for 64% of the proven carriers. When excluding the two most common variants (c.67G>C and c.225_243del), PDAC was observed in 2/12 (17%) families with a variant affecting both proteins, and in 5/11 (45%) families with a variant affecting solely p16INK4a. Combining all variants with an effect on p16INK4a, regardless of the effect on p14ARF, PDAC occurred in 95/163 (58%) families that included 373/629 (59%) proven carriers. By contrast, PDAC was not observed in the nine families (20 proven carriers) with a variant solely affecting the p14ARF protein.

### *CDKN2A* c.67G>C variant and pancreatic cancer risk

The c.67G>C, p.(Gly23Arg) variant was found in nine families and is therefore the second most prevalent pathogenic *CDKN2A* variant found in Dutch melanoma families (table 1 and figure 1). Together, these families comprised 251 at-risk family members (84 proven and obligate carriers, 90 individuals with an a priori 50% chance of carriership and 77 with an a priori 25% chance; table 2). During a total follow-up of 10 414 person-years, PDAC developed in 12 individuals (9 were carriers, including 2 PDAC index cases; 2 had a 50% chance of carriership; 1 a 25% chance). PDAC did not develop in the 52 proven non-carriers.

**Table 2:** Details of the families in which the c.67G>C, p.(Gly23Arg) variant was found

Family number	Total number of family members at risk*	Carriers†	50% chance of carriership	25% chance of carriership	Proven non-carriers	No. of PDAC cases in carriers† and possible carriers (of which validated‡)
1	11	7	4	0	3	0 (−)
2	56	9	17	30	5	3 (1)
3	44	11	19	14	7	1 (1)
4	26	9	7	10	6	0 (−)
5	8	6	2	0	9	4 (2)
6	53	24	21	8	16	2 (0)
7	19	6	7	6	0	1 (1)
8	14	9	5	0	5	0 (−)
9	20	3	8	9	1	1 (0)
Total	251	84	90	77	52	12

*Proven non-carriers excluded.

†Includes proven and obligate carriers.

‡Through medical records and/or pathology reports.

PDAC, pancreatic ductal adenocarcinoma.

**Figure 2:**
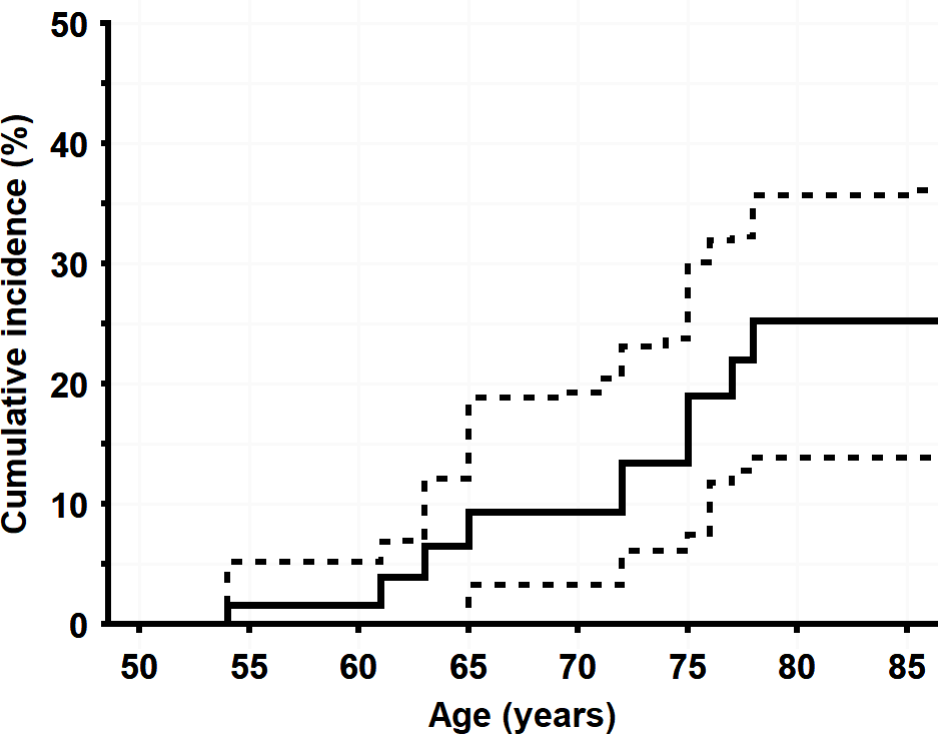
Cumulative pancreatic cancer incidence for c.67G>C, p.(Gly23Arg) carriers, with 95% CIs shown as dashed lines.

After excluding the two PDAC index cases and the family members with a 25% chance of carriership, the ratio of observed/expected cases was 9/0.47, resulting in a SIR of 19.1 (95% CI 8.3 to 33.6). The cumulative PDAC incidence for carriers of the c.67G>C, p.(Gly23Arg) variant is shown in figure 2. At age 75 years, the cumulative PDAC incidence (lifetime risk) was 19% (95% CI 7.5% to 30.1%).

## Discussion

The precise relationship between the *CDKN2A* gene and PDAC is unclear and an association has only been confirmed for a subset of pathogenic variants.^19^ In order to properly counsel carriers and to determine whether they are eligible for pancreatic cancer surveillance, a better understanding of possible genotype-phenotype correlations is needed. In this study, we investigated PDAC occurrence in a Dutch nationwide cohort of melanoma families with a (likely) pathogenic *CDKN2A* germline variant, and estimated the lifetime risk associated with the variant c.67G>C, p.(Gly23Arg). Of the 15 different variants, the majority (87%) affected the p16INK4a protein and PDAC occurred in 58% of families harbouring these variants. By contrast, we observed no PDAC in carriers (or their family members) of the two variants (both in exon 1β) that solely affected the p14ARF protein (9 families, 20 carriers). This outcome supports the findings of earlier studies, which are summarised in online supplementary tables 1 and 2. To date, a total of 35 families with exon 1β variants (including the 9 families in our current study) have been reported worldwide, and PDAC has not been reported in any of them (see online supplementary table 1). A recent meta-analysis also failed to find an association between PDAC and pathogenic variants in exon 1β.^19^ In our cohort, all variants in exon 2 impaired both proteins, but other studies have described patients with melanoma and families with exon 2 variants altering only one of the two proteins. These studies have reported PDAC occurrence for exon 2 variants affecting only p16INK4a,^6^ but there are no such reports for exon 2 variants solely altering p14ARF (see online supplementary table 2). Of note, in a study by Kannengiesser *et al*,^28^ PDAC was reported in two out of four melanoma families with the c.339G>A variant that solely affects p14ARF, but as this variant is co-occurring in these families with a second variant that does alter p16INK4A (c.340C>T), they cannot be considered as sole p14ARF-families. Thus, although variants affecting only p14ARF have been described in a small number of families, these earlier observations together with our own findings strongly suggest that impairment of only the p14ARF protein (and therefore the p14ARF-p53 pathway) is not associated with an increased PDAC risk. Of the individual variants in our cohort, the most prevalent was the p16-*Leiden* variant (c.225_243del, p.(Ala76Cysfs*64)), which was present in the majority (76%) of families, in many of which (63%) PDAC occurred. This is unsurprising, as earlier studies have described the high prevalence of this variant in the Netherlands and its strong association with PDAC.^8 10^ Yet, other pathogenic variants were also found regularly (in 24% of families), the most prevalent was c.67G>C, p.(Gly23Arg). Single families harbouring this variant^29 30^ or other variants within the same codon^29–35^ have been previously described, mainly in the context of melanoma risk. Of these studies only one also reported on PDAC occurrence, describing a single case in a second-degree relative of a c.67G>A, p.(Gly23Ser) variant carrier.^34^ Based on our larger set of nine families, we estimated the lifetime PDAC risk associated with this specific variant to be approximately equivalent to that of the p16-*Leiden* variant (estimated at 15%–20%).^8 10^

10.1136/jmedgenet-2019-106562.supp1 Supplementary data

Of the remaining 13 variants, 10 have been found in families in other countries,^7 19 36^ but 3 have not been previously described in literature. These were c.143C>A (located on a pathogenic haplotype), c.151-2A>G and a deletion of 155 kb spanning *CDKN2A*, *CDKN2B* and a part of *MTAP*. Two of these variants (c.143C>A and c.151-2A>G) were identified in families with one or more occurrences of PDAC. Since the majority of variants affecting p16INK4a in our study were identified in melanoma families with one or more occurrences of PDAC, and most other variants affecting p16INK4a have been associated with a positive family history for PDAC in previous studies (table 1), our results contribute to a growing body of evidence that suggests that all pathogenic variants affecting the p16INK4a protein convey an increased PDAC risk. This trend is further supported by the risk estimates for the two most prevalent variants in the Netherlands, both of which affect p16INK4a and exceed the 5% threshold defined by the international cancer of the pancreas surveillance consortium.^17^ Although we consider it likely that most other pathogenic variants affecting p16INK4a will exceed the 5% lifetime risk threshold for PDAC surveillance as well, we could not specifically investigate this for every single variant in our cohort due to their low prevalence. We do acknowledge that somewhat lower frequencies of PDAC have previously been reported in families harbouring variants in specific domains of the *CDKN2A* gene, that is, the ankyrin repeats 1 and 2 (corresponding to codons 11–71 of the p16INK4a transcript),^6 7 37^ but our data do not support this possible genotype-phenotype correlation between variants in specific ankyrin repeats and PDAC risk since the c.67G>C, p.(Gly23Arg) variant lies within the ankyrin repeat 1 and has shown a particular strong association with PDAC risk. Therefore, based on existing literature and this study, all carriers of pathogenic *CDKN2A* variants affecting p16INK4a should be considered candidates for PDAC surveillance, until convincing evidence to the contrary emerges. Conversely, available evidence does not support an increased PDAC risk in carriers of variants solely affecting p14ARF, and carriers of these variants should, therefore, not be offered surveillance at this time. Our study had several strengths. The centralised Dutch database for families harbouring (likely) pathogenic *CDKN2A* variants allowed us to examine the complete spectrum of variants and the occurrence of PDAC on a national level. This facilitated estimation of the lifetime PDAC risk for the second most prevalent variant, enabling better genetic counselling. Second, we also corrected for possible ascertainment bias by excluding all PDAC index cases from the analysis, thereby avoiding risk overestimation. Third, it is unlikely that a recall or reporting bias substantially influenced our results. To minimise this risk, we excluded all untested second-degree and higher-degree family members (with and without PDAC) from the analyses. Furthermore, it has been shown that reported family histories have a near perfect (>99%) negative predictive value for PDAC,^38^ and a high positive predictive value for PDAC diagnoses in first-degree relatives (>75%).^26^

One limitation of our study should be noted. Notwithstanding the large number of families included, for each individual variant the number of families, variant carriers and PDAC cases was low. Besides the previously described p16-*Leiden* variant, the c.67G >C, p.(Gly23Arg) variant was the only variant for which we had sufficient PDAC cases to calculate lifetime risk estimates. Clearly, achieving reliable future estimates of lifetime PDAC risk for most variants will require international reporting and the amalgamation of results on variant frequencies and PDAC occurrence.

In conclusion, a variety of pathogenic *CDKN2A* variants other than the relatively prevalent p16-*Leiden* variant are found in Dutch melanoma families. Pathogenic variants affecting p16INK4a (including c.67G>C, p.(Gly23Arg)), but not those affecting only p14ARF, appear to be associated with increased PDAC risk. Therefore, carriers of such variants should be eligible for pancreatic cancer surveillance programmes within a research setting.

## References

[1] Aoude LG , Wadt KAW , Pritchard AL , Hayward NK . Genetics of familial melanoma: 20 years after CDKN2A. Pigment Cell Melanoma Res 2015;28:148–60. 10.1111/pcmr.12333 25431349

[2] Sherr CJ . The INK4a/ARF network in tumour suppression. Nat Rev Mol Cell Biol 2001;2:731–7. 10.1038/35096061 11584300

[3] Bishop DT , Demenais F , Goldstein AM , Bergman W , Bishop JN , Bressac-de Paillerets B , Chompret A , Ghiorzo P , Gruis N , Hansson J , Harland M , Hayward N , Holland EA , Mann GJ , Mantelli M , Nancarrow D , Platz A , Tucker MA , Melanoma Genetics C , Melanoma Genetics Consortium. Geographical variation in the penetrance of CDKN2A mutations for melanoma. J Natl Cancer Inst 2002;94:894–903. 10.1093/jnci/94.12.894 12072543

[4] Read J , Wadt KAW , Hayward NK . Melanoma genetics. J Med Genet 2016;53:1–14. 10.1136/jmedgenet-2015-103150 26337759

[5] Tucker MA , Elder DE , Curry M , Fraser MC , Pichler V , Zametkin D , Yang XR , Goldstein AM . Risks of melanoma and other cancers in melanoma-prone families over 4 decades. J Invest Dermatol 2018;138:1620–6. 10.1016/j.jid.2018.01.021 29408205 PMC7021443

[6] Goldstein AM . Familial melanoma, pancreatic cancer and germline CDKN2A mutations. Hum Mutat 2004;23:630. 10.1002/humu.9247 15146471

[7] Goldstein AM , Chan M , Harland M , Gillanders EM , Hayward NK , Avril M-F , Azizi E , Bianchi-Scarra G , Bishop DT , Bressac-de Paillerets B , Bruno W , Calista D , Cannon Albright LA , Demenais F , Elder DE , Ghiorzo P , Gruis NA , Hansson J , Hogg D , Holland EA , Kanetsky PA , Kefford RF , Landi MT , Lang J , Leachman SA , MacKie RM , Magnusson V , Mann GJ , Niendorf K , Newton Bishop J , Palmer JM , Puig S , Puig-Butille JA , de Snoo FA , Stark M , Tsao H , Tucker MA , Whitaker L , Yakobson E , the Melanoma Genetics Consortium (GenoMEL) 26. High-risk melanoma susceptibility genes and pancreatic cancer, neural system tumors, and uveal melanoma across GenoMEL. Cancer Res 2006;66:9818–28. 10.1158/0008-5472.CAN-06-0494 17047042

[8] de Snoo FA , Bishop DT , Bergman W , van Leeuwen I , van der Drift C , van Nieuwpoort FA , Out-Luiting CJ , Vasen HF , ter Huurne JAC , Frants RR , Willemze R , Breuning MH , Gruis NA . Increased risk of cancer other than melanoma in CDKN2A founder mutation (p16-Leiden)-positive melanoma families. Clin Cancer Res 2008;14:7151–7. 10.1158/1078-0432.CCR-08-0403 18981015

[9] Goldstein AM , Fraser MC , Struewing JP , Hussussian CJ , Ranade K , Zametkin DP , Fontaine LS , Organic SM , Dracopoli NC , Clark WH . Increased risk of pancreatic cancer in melanoma-prone kindreds with p16INK4 mutations. N Engl J Med 1995;333:970–5. 10.1056/NEJM199510123331504 7666916

[10] Vasen HF , Gruis NA , Frants RR , van Der Velden PA , Hille ET , Bergman W . Risk of developing pancreatic cancer in families with familial atypical multiple mole melanoma associated with a specific 19 deletion of p16 (p16-Leiden). Int J Cancer 2000;87:809–11. 10.1002/1097-0215(20000915)87:6&lt;809::AID-IJC8&gt;3.0.CO;2-U 10956390

[11] Vasen H , Ibrahim I , Ponce CG , Slater EP , Matthäi E , Carrato A , Earl J , Robbers K , van Mil AM , Potjer T , Bonsing BA , de Vos Tot Nederveen Cappel WH , Bergman W , Wasser M , Klöppel G , Klöppel G , Schicker C , Steinkamp M , Figiel J , Esposito I , Mocci E , Vazquez-Sequeiros E , Muñoz-Beltran M , Muñoz-Beltran M , Montans J , Langer P , Fendrich V , Bartsch DK . Benefit of surveillance for pancreatic cancer in high-risk individuals: outcome of long-term prospective follow-up studies from three European expert centers. J Clin Oncol 2016;34:2010–9. 10.1200/JCO.2015.64.0730 27114589

[12] Hu C , Hart SN , Polley EC , Gnanaolivu R , Shimelis H , Lee KY , Lilyquist J , Na J , Moore R , Antwi SO , Bamlet WR , Chaffee KG , DiCarlo J , Wu Z , Samara R , Kasi PM , McWilliams RR , Petersen GM , Couch FJ . Association between inherited germline mutations in cancer predisposition genes and risk of pancreatic cancer. JAMA 2018;319:2401–9. 10.1001/jama.2018.6228 29922827 PMC6092184

[13] Overbeek KA , Cahen DL , Canto MI , Bruno MJ . Surveillance for neoplasia in the pancreas. Best Pract Res Clin Gastroenterol 2016;30:971–86. 10.1016/j.bpg.2016.10.013 27938791 PMC5552042

[14] Ghiorzo P , Fornarini G , Sciallero S , Battistuzzi L , Belli F , Bernard L , Bonelli L , Borgonovo G , Bruno W , De Cian F , Decensi A , Filauro M , Faravelli F , Gozza A , Gargiulo S , Mariette F , Nasti S , Pastorino L , Queirolo P , Savarino V , Varesco L , Scarrà GB , Genoa Pancreatic Cancer Study Group. CDKN2A is the main susceptibility gene in Italian pancreatic cancer families. J Med Genet 2012;49:164–70. 10.1136/jmedgenet-2011-100281 22368299

[15] Zhen DB , Rabe KG , Gallinger S , Syngal S , Schwartz AG , Goggins MG , Hruban RH , Cote ML , McWilliams RR , Roberts NJ , Cannon-Albright LA , Li D , Moyes K , Wenstrup RJ , Hartman A-R , Seminara D , Klein AP , Petersen GM . BRCA1, BRCA2, PALB2, and CDKN2A mutations in familial pancreatic cancer: a PACGENE study. Genet Med 2015;17:569–77. 10.1038/gim.2014.153 25356972 PMC4439391

[16] Harinck F , Kluijt I , van der Stoep N , Oldenburg RA , Wagner A , Aalfs CM , Sijmons RH , Poley J-W , Kuipers EJ , Fockens P , van Os TAM , Bruno MJ . Indication for CDKN2A-mutation analysis in familial pancreatic cancer families without melanomas. J Med Genet 2012;49:362–5. 10.1136/jmedgenet-2011-100563 22636603

[17] Goggins M , Overbeek KA , Brand R , Syngal S , Del Chiaro M , Bartsch DK , Bassi C , Carrato A , Farrell J , Fishman EK , Fockens P , Gress TM , van Hooft JE , Hruban RH , Kastrinos F , Klein A , Lennon AM , Lucas A , Park W , Rustgi A , Simeone D , Stoffel E , Vasen HFA , Cahen DL , Canto MI , Bruno M , International Cancer of the Pancreas Screening (CAPS) consortium. Management of patients with increased risk for familial pancreatic cancer: updated recommendations from the International cancer of the pancreas screening (CAPS) Consortium. Gut 2020;69:7–17. 10.1136/gutjnl-2019-319352 31672839 PMC7295005

[18] Goldstein AM , Struewing JP , Chidambaram A , Fraser MC , Tucker MA . Genotype-phenotype relationships in U.S. melanoma-prone families with CDKN2A and CDK4 mutations. J Natl Cancer Inst 2000;92:1006–10. 10.1093/jnci/92.12.1006 10861313

[19] Zhan W , Shelton CA , Greer PJ , Brand RE , Whitcomb DC . Germline variants and risk for pancreatic cancer: a systematic review and emerging concepts. Pancreas 2018;47:924–36. 10.1097/MPA.0000000000001136 30113427 PMC6097243

[20] Gruis NA , van der Velden PA , Sandkuijl LA , Prins DE , Weaver-Feldhaus J , Kamb A , Bergman W , Frants RR . Homozygotes for CDKN2 (p16) germline mutation in Dutch familial melanoma kindreds. Nat Genet 1995;10:351–3. 10.1038/ng0795-351 7670475

[21] Goldstein AM , Chan M , Harland M , Gillanders EM , Hayward NK , Avril M-F , Azizi E , Bianchi-Scarra G , Bishop DT , Bressac-de Paillerets B , Bruno W , Calista D , Cannon Albright LA , Demenais F , Elder DE , Ghiorzo P , Gruis NA , Hansson J , Hogg D , Holland EA , Kanetsky PA , Kefford RF , Landi MT , Lang J , Leachman SA , Mackie RM , Magnusson V , Mann GJ , Niendorf K , Newton Bishop J , Palmer JM , Puig S , Puig-Butille JA , de Snoo FA , Stark M , Tsao H , Tucker MA , Whitaker L , Yakobson E , Melanoma Genetics C , Melanoma Genetics Consortium (GenoMEL). High-risk melanoma susceptibility genes and pancreatic cancer, neural system tumors, and uveal melanoma across GenoMEL. Cancer Res 2006;66:9818–28. 10.1158/0008-5472.CAN-06-0494 17047042

[22] Potjer TP , Helgadottir H , Leenheer M , van der Stoep N , Gruis NA , Hoiom V , Olsson H , van Doorn R , Vasen HFA , van Asperen CJ , Dekkers OM , Hes FJ . Dutch Working group for clinical O. CM-Score: a validated scoring system to predict CDKN2A germline mutations in melanoma families from northern Europe. J Med Genet 2018;55:661–8. 29661971 10.1136/jmedgenet-2017-105205

[23] Plon SE , Eccles DM , Easton D , Foulkes WD , Genuardi M , Greenblatt MS , Hogervorst FBL , Hoogerbrugge N , Spurdle AB , Tavtigian SV , IARC Unclassified Genetic Variants Working Group. Sequence variant classification and reporting: recommendations for improving the interpretation of cancer susceptibility genetic test results. Hum Mutat 2008;29:1282–91. 10.1002/humu.20880 18951446 PMC3075918

[24] Pollock PM , Spurr N , Bishop T , Newton-Bishop J , Gruis N , van der Velden PA , Goldstein AM , Tucker MA , Foulkes WD , Barnhill R , Haber D , Fountain J , Hayward NK . Haplotype analysis of two recurrent CDKN2A mutations in 10 melanoma families: evidence for common founders and independent mutations. Hum Mutat 1998;11:424–31. 10.1002/(SICI)1098-1004(1998)11:6&lt;424::AID-HUMU2&gt;3.0.CO;2-2 9603434

[25] Yakobson E , Eisenberg S , Isacson R , Halle D , Levy-Lahad E , Catane R , Safro M , Sobolev V , Huot T , Peters G , Ruiz A , Malvehy J , Puig S , Chompret A , Avril M-F , Shafir R , Peretz H , Bressac-de Paillerets B . A single Mediterranean, possibly Jewish, origin for the Val59Gly CDKN2A mutation in four melanoma-prone families. Eur J Hum Genet 2003;11:288–96. 10.1038/sj.ejhg.5200961 12700603

[26] Fiederling J , Shams AZ , Haug U . Validity of self-reported family history of cancer: a systematic literature review on selected cancers. Int J Cancer 2016;139:1449–60. 10.1002/ijc.30203 27222437

[27] Dutch Cancer Registration (NKR) I. [Web Page]. Netherlands Comprehensive Cancer Organisation [cited 18 November 2019], 2018. Available: www.cijfersoverkanker.nl

[28] Kannengiesser C , Brookes S , del Arroyo AG , Pham D , Bombled J , Barrois M , Mauffret O , Avril M-FM , Chompret A , Lenoir GM , Sarasin A , Peters G , Bressac-de Paillerets B , French Hereditary Melanoma Study Group. Functional, structural, and genetic evaluation of 20 CDKN2A germ line mutations identified in melanoma-prone families or patients. Hum Mutat 2009;30:564–74. 10.1002/humu.20845 19260062

[29] Bruno W , Pastorino L , Ghiorzo P , Andreotti V , Martinuzzi C , Menin C , Elefanti L , Stagni C , Vecchiato A , Rodolfo M , Maurichi A , Manoukian S , De Giorgi V , Savarese I , Gensini F , Borgognoni L , Testori A , Spadola G , Mandalà M , Imberti G , Savoia P , Astrua C , Ronco AM , Farnetti A , Tibiletti MG , Lombardo M , Palmieri G , Ayala F , Ascierto P , Ghigliotti G , Muggianu M , Spagnolo F , Picasso V , Tanda ET , Queirolo P , Bianchi-Scarrà G . Multiple primary melanomas (MPMs) and criteria for genetic assessment: MultiMEL, a multicenter study of the Italian melanoma intergroup. J Am Acad Dermatol 2016;74:325–32. 10.1016/j.jaad.2015.09.053 26775776

[30] Berwick M , Orlow I , Hummer AJ , Armstrong BK , Kricker A , Marrett LD , Millikan RC , Gruber SB , Anton-Culver H , Zanetti R , Gallagher RP , Dwyer T , Rebbeck TR , Kanetsky PA , Busam K , From L , Mujumdar U , Wilcox H , Begg CB , GEM Study Group. The prevalence of CDKN2A germ-line mutations and relative risk for cutaneous malignant melanoma: an international population-based study. Cancer Epidemiol Biomarkers Prev 2006;15:1520–5. 10.1158/1055-9965.EPI-06-0270 16896043

[31] Soufir N , Avril MF , Chompret A , Demenais F , Bombled J , Spatz A , Stoppa-Lyonnet D , Bénard J , Bressac-de Paillerets B . Prevalence of p16 and CDK4 germline mutations in 48 melanoma-prone families in France. The French familial melanoma Study Group. Hum Mol Genet 1998;7:209–16. 10.1093/hmg/7.2.209 9425228

[32] Fargnoli MC , Chimenti S , Keller G , Soyer HP , Dal Pozzo V , Höfler H , Peris K . CDKN2a/p16INK4a mutations and lack of p19ARF involvement in familial melanoma kindreds. J Invest Dermatol 1998;111:1202–6. 10.1046/j.1523-1747.1998.00412.x 9856841

[33] Blackwood MA , Holmes R , Synnestvedt M , Young M , George C , Yang H , Elder DE , Schuchter LM , Guerry D , Ganguly A . Multiple primary melanoma revisited. Cancer 2002;94:2248–55. 10.1002/cncr.10454 12001124

[34] Gensini F , Sestini R , Piazzini M , Vignoli M , Chiarugi A , Brandani P , Ghiorzo P , Salvini C , Borgognoni L , Palli D , Bianchi-Scarrà G , Carli P , Genuardi M . The p.G23S CDKN2A founder mutation in high-risk melanoma families from central Italy. Melanoma Res 2007;17:387–92. 10.1097/CMR.0b013e3282f1d328 17992122

[35] Scaini MC , Rossi E , de Siqueira Torres PLA , Zullato D , Callegaro M , Casella C , Quaggio M , Agata S , Malacrida S , Chiarion-Sileni V , Vecchiato A , Alaibac M , Montagna M , Mann GJ , Menin C , D'Andrea E . Functional impairment of p16(INK4A) due to CDKN2A p.Gly23Asp missense mutation. Mutat Res 2009;671:26–32. 10.1016/j.mrfmmm.2009.08.007 19712690

[36] Gerdes B , Bartsch DK , Ramaswamy A , Kersting M , Wild A , Schuermann M , Frey M , Rothmund M . Multiple primary tumors as an indicator for p16INK4a germline mutations in pancreatic cancer patients? Pancreas 2000;21:369–75. 10.1097/00006676-200011000-00007 11075991

[37] Goldstein AM , Chan M , Harland M , Hayward NK , Demenais F , Bishop DT , Azizi E , Bergman W , Bianchi-Scarra G , Bruno W , Calista D , Albright LAC , Chaudru V , Chompret A , Cuellar F , Elder DE , Ghiorzo P , Gillanders EM , Gruis NA , Hansson J , Hogg D , Holland EA , Kanetsky PA , Kefford RF , Landi MT , Lang J , Leachman SA , MacKie RM , Magnusson V , Mann GJ , Bishop JN , Palmer JM , Puig S , Puig-Butille JA , Stark M , Tsao H , Tucker MA , Whitaker L , Yakobson E , Lund Melanoma Study Group, Melanoma Genetics Consortium (GenoMEL). Features associated with germline CDKN2A mutations: a GenoMEL study of melanoma-prone families from three continents. J Med Genet 2007;44:99–106. 10.1136/jmg.2006.043802 16905682 PMC2598064

[38] Ziogas A , Anton-Culver H . Validation of family history data in cancer family registries. Am J Prev Med 2003;24:190–8. 10.1016/S0749-3797(02)00593-7 12568826

