# Supplementary material for: Genotype-phenotype correlations for pancreatic cancer risk in Dutch melanoma families with pathogenic CDKN2A variants
Source: J Med Genet. 2020 Jun 1;58(4):264–9. doi: 10.1136/jmedgenet-2019-106562 (PMC8005797; doi:10.1136/jmedgenet-2019-106562)
Supplement: Supplementary data [file jmedgenet-2019-106562supp001.pdf]

SUPPLEMENTARY TABLE 1. Overview of published families with variants in exon 1β.

CDKN2A nucleotide change (exon 1β)†	*CDKN2A* / p14ARF amino acid change	Total no. of families	PDAC reported in families	References
c.45_60dup	p.(Val22Profs*46)	1	Not described	[1]
c.47G>A	p.(Gly16Asp)	1	No	[2]
c.60ins16	insertion	1	No	[3]
c.62G>A	p.(Arg21Lys)	1‡	No	[4]
c.92C>T	p.(Thr31Met)	1	No	[5]
c.81C>G	p.(Ile27Met)	1	Not described	[1]
c.102G>A	p.(Trp34*)	1	Not described	[1]
c.161G>A	p.(Arg54His)	1	No	[6, 7]
c.192A>T	p.(Pro64Pro) (splicing)	1	No	[8, 9]
c.193G>C	p.(Gly65Arg)	8‡, ¶	No (7)<br>Not described (1)	[1, 8, 10-12]
c.193G>A	p.(Gly65Ser)	2	No (1)<br>Not described (1)	[1, 13]
c.193+1G>C	p.? (splicing)	Not described	Not described	[14]
c.193+1G>A	p.? (splicing)	9‡, §	No (8)<br>Not described (1)	[6-9, 12, 15, 16]
c.193+2T>C	p.? (splicing)	1‡	No	[8, 9]
193+3A>G	p.? (splicing)	1‡	No	[8, 9]
c.193+5G>A	p.? (splicing)	2‡	No	[4]
deletion exon 1β	deletion exon 1β	3‡	No	[2, 9, 17]
**Total**		**35**	**No (29)**<br>**Not described (6)**	

† RefSeq NM_058195.3 isoform p14ARF.
‡ Variant is also reported in Taylor *et al* 2016[18] and Taylor *et al* 2017[14], but the number of families in which the variant was found was not described (and possibly overlaps with other studies).
¶ Includes 4 families that were reported by Potjer *et al* 2019[12] and are included in the current study.
§ Includes 5 families that were reported by Potjer *et al* 2019[12] and are included in the current study.

Overbeek KA, *et al. J Med Genet* 2021; 58:264–269. doi: 10.1136/jmedgenet-2019-106562

SUPPLEMENTARY TABLE 2. Overview of published families with variants in exon 2 that solely affect p14ARF.

CDKN2A nucleotide change (exon 2)†	CDKN2A / p16INK4a amino acid change	CDKN2A / p14ARF amino acid change	Total no. of families	PDAC reported in families	References
c.174A>C	p.(Arg58=)	p.(Ser73Arg)	1	Not described	[19-21]
c.192G>A	p.(Leu64=)	p.(Ala79Thr)	1‡	Not described	[14, 19, 22]
c.192G>C	p.(Leu64=)	p.(Ala79Pro)	1	Found in sporadic PDAC patient	[23, 24]
c.198C>T	p.(His66=)	p.(Arg81Trp)	1‡	No	[14, 18, 25]
c.225C>T	p.(Pro75=)	p.(Arg90Cys)	1	Found in sporadic PDAC patient	[24]
c.267C>A	p.(Gly89=)	p.(Leu104Ile)	1	Not described	[22]
c.294C>T	p.(His98=)	p.(Pro113Ser)	2	Not described	[16]
c.306G>A	p.(Ala102=)	p.(Ala117Thr)	1	Not described	[19, 21]
c.318G>A	p.(Val106=)	p.(Ala121Thr)	12‡	Found in sporadic PDAC patient (1)<br>No (4)<br>Not described (7)	[1, 14, 18-24, 26-28]
c.339G>A	p.(Leu113=)	p.(Ala128Thr)	8	Not described (4)<br>No (2)¶<br>Yes (2)¶	[9, 16, 22, 29-31]
c.339G>C	p.(Leu113=)	p.(Ala128Pro)	Not described	Not described	[14, 18]
**Total**			**29**	**No (7)**<br>**Not described (17)**<br>**Found in sporadic PDAC (3)**<br>**Yes (2)**	

† RefSeq NM_000077.4 isoform p16INK4a
‡ Variant is also reported in Taylor *et al* 2016[18] and Taylor *et al* 2017[14], but the number of families in which the variant was found was not described (and possibly overlaps with other studies).
¶ In the families reported by Kannengiesser *et al* 2009[30], the variant c.339G>C co-occurred with variant c.340C>T which has an effect on p16INK4a (p.Pro114Ser)

REFERENCES

1. Harland M, Cust AE, Badenas C, Chang YM, Holland EA, Aguilera P, Aitken JF, Armstrong BK, Barrett JH, Carrera C, Chan M, Gascoyne J, Giles GG, Agha-Hamilton C, Hopper JL, Jenkins MA, Kanetsky PA, Kefford RF, Kolm I, Lowery J, Malvehy J, Ogbah Z, Puig-Butille JA, Orihuela-Segales J, Randerson-Moor JA, Schmid H, Taylor CF, Whitaker L, Bishop DT, Mann GJ, Newton-Bishop JA, Puig S (2014) Prevalence and predictors of germline CDKN2A mutations for melanoma cases from Australia, Spain and the United Kingdom. Hered Cancer Clin Pract 12(1):20. 10.1186/1897-4287-12-20
2. Laud K, Marian C, Avril MF, Barrois M, Chompret A, Goldstein AM, Tucker MA, Clark PA, Peters G, Chaudru V, Demenais F, Spatz A, Smith MW, Lenoir GM, Bressac-de Paillerets B, French Hereditary Melanoma Study G (2006) Comprehensive analysis of CDKN2A (p16INK4A/p14ARF) and CDKN2B genes in 53 melanoma index cases considered to be at heightened risk of melanoma. J Med Genet 43(1):39-47. 10.1136/jmg.2005.033498
3. Rizos H, Puig S, Badenas C, Malvehy J, Darmanian AP, Jimenez L, Mila M, Kefford RF (2001) A melanoma-associated germline mutation in exon 1beta inactivates p14ARF. Oncogene 20(39):5543-7. 10.1038/sj.onc.1204728
4. Wadt KA, Aoude LG, Krogh L, Sunde L, Bojesen A, Gronskov K, Wartacz N, Ek J, Tolstrup-Andersen M, Klarskov-Andersen M, Borg A, Heegaard S, Kiilgaard JF, Hansen TV, Klein K, Jonsson G, Drzewiecki KT, Duno M, Hayward NK, Gerdes AM (2015) Molecular characterization of melanoma cases in Denmark suspected of genetic predisposition. PLoS One 10(3):e0122662. 10.1371/journal.pone.0122662
5. Pellegrini C, Maturo MG, Martorelli C, Suppa M, Antonini A, Kostaki D, Verna L, Landi MT, Peris K, Fargnoli MC (2017) Characterization of melanoma susceptibility genes in high-risk patients from Central Italy. Melanoma Res 27(3):258-267. 10.1097/CMR.0000000000000323
6. Binni F, Antigoni I, De Simone P, Majore S, Silipo V, Crisi A, Amantea A, Pacchiarini D, Castori M, De Bernardo C, Catricala C, Grammatico P (2010) Novel and recurrent p14 mutations in Italian familial melanoma. Clin Genet 77(6):581-6. 10.1111/j.1399-0004.2009.01298.x
7. Pedace L, De Simone P, Castori M, Sperduti I, Silipo V, Eibenschutz L, De Bernardo C, Buccini P, Moscarella E, Panetta C, Ferrari A, Grammatico P, Catricala C (2011) Clinical features predicting identification of CDKN2A mutations in Italian patients with familial cutaneous melanoma. Cancer Epidemiol 35(6):e116-20. 10.1016/j.canep.2011.07.007
8. Harland M, Taylor CF, Chambers PA, Kukalizch K, Randerson-Moor JA, Gruis NA, de Snoo FA, ter Huurne JA, Goldstein AM, Tucker MA, Bishop DT, Bishop JA (2005) A mutation hotspot at the p14ARF splice site. Oncogene 24(28):4604-8. 10.1038/sj.onc.1208678
9. Goldstein AM, Chan M, Harland M, Gillanders EM, Hayward NK, Avril MF, Azizi E, Bianchi-Scarra G, Bishop DT, Bressac-de Paillerets B, Bruno W, Calista D, Cannon Albright LA, Demenais F, Elder DE, Ghiorzo P, Gruis NA, Hansson J, Hogg D, Holland EA, Kanetsky PA, Kefford RF, Landi MT, Lang J, Leachman SA, Mackie RM, Magnusson V, Mann GJ, Niendorf K, Newton Bishop J, Palmer JM, Puig S, Puig-Butille JA, de Snoo FA, Stark M, Tsao H, Tucker MA, Whitaker L, Yakobson E (2006) High-risk melanoma susceptibility genes and pancreatic cancer, neural system tumors, and uveal melanoma across GenoMEL. Cancer Res 66(20):9818-28. 10.1158/0008-5472.can-06-0494
10. Hewitt C, Lee Wu C, Evans G, Howell A, Elles RG, Jordan R, Sloan P, Read AP, Thakker N (2002) Germline mutation of ARF in a melanoma kindred. Hum Mol Genet 11(11):1273-9. 10.1093/hmg/11.11.1273
11. Garcia-Casado Z, Nagore E, Fernandez-Serra A, Botella-Estrada R, Lopez-Guerrero JA (2009) A germline mutation of p14/ARF in a melanoma kindred. Melanoma Res 19(5):335-7. 10.1097/CMR.0b013e32832dd2d4
12. Potjer TP, Helgadottir H, Leenheer M, van der Stoep N, Gruis NA, Hoiom V, Olsson H, van Doorn R, Vasen HFA, van Asperen CJ, Dekkers OM, Hes FJ, Dutch Working Group for Clinical O (2018) CM-Score: a validated scoring system to predict CDKN2A germline mutations in melanoma families from Northern Europe. J Med Genet 55(10):661-668. 10.1136/jmedgenet-2017-105205
13. McInerney-Leo AM, Wheeler L, Sturm RA, Tan JM, Harris JE, Anderson L, Jagirdar K, Brown MA, Leo PJ, Soyer HP, Duncan EL (2018) Point mutation in p14(ARF) -specific exon 1beta of

CDKN2A causing familial melanoma and astrocytoma. Br J Dermatol 178(4):e263-e264. 10.1111/bjd.16275

14. Taylor NJ, Mitra N, Goldstein AM, Tucker MA, Avril MF, Azizi E, Bergman W, Bishop DT, Bressac-de Paillerets B, Bruno W, Calista D, Cannon-Albright LA, Cuellar F, Cust AE, Demenais F, Elder DE, Gerdes AM, Ghiorzo P, Grazziotin TC, Hansson J, Harland M, Hayward NK, Hocevar M, Hoiom V, Ingvar C, Landi MT, Landman G, Larre-Borges A, Leachman SA, Mann GJ, Nagore E, Olsson H, Palmer JM, Peric B, Pjanova D, Pritchard A, Puig S, van der Stoep N, Wadt KAW, Whitaker L, Yang XR, Newton Bishop JA, Gruis NA, Kanetsky PA, Geno MELSG (2017) Germline Variation at CDKN2A and Associations with Nevus Phenotypes among Members of Melanoma Families. J Invest Dermatol 137(12):2606-2612. 10.1016/j.jid.2017.07.829
15. Ghiorzo P, Pastorino L, Pizzichetta MA, Bono R, Queirolo P, Talamini R, Annessi G, Bruno W, Nasti S, Gargiulo S, Battistuzzi L, Sini MC, Palmieri G, Scarra GB, Italian Melanoma I (2009) CDKN2A and MC1R analysis in amelanotic and pigmented melanoma. Melanoma Res 19(3):142-5. 10.1097/CMR.0b013e32832a1e18
16. Bruno W, Pastorino L, Ghiorzo P, Andreotti V, Martinuzzi C, Menin C, Elefanti L, Stagni C, Vecchiato A, Rodolfo M, Maurichi A, Manoukian S, De Giorgi V, Savarese I, Gensini F, Borgognoni L, Testori A, Spadola G, Mandala M, Imberti G, Savoia P, Astrua C, Ronco AM, Farnetti A, Tibiletti MG, Lombardo M, Palmieri G, Ayala F, Ascierto P, Ghigliotti G, Muggianu M, Spagnolo F, Picasso V, Tanda ET, Queirolo P, Bianchi-Scarra G (2016) Multiple primary melanomas (MPMs) and criteria for genetic assessment: MultiMEL, a multicenter study of the Italian Melanoma Intergroup. J Am Acad Dermatol 74(2):325-32. 10.1016/j.jaad.2015.09.053
17. Randerson-Moor JA, Harland M, Williams S, Cuthbert-Heavens D, Sheridan E, Aveyard J, Sibley K, Whitaker L, Knowles M, Bishop JN, Bishop DT (2001) A germline deletion of p14(ARF) but not CDKN2A in a melanoma-neural system tumour syndrome family. Hum Mol Genet 10(1):55-62. 10.1093/hmg/10.1.55
18. Taylor NJ, Handorf EA, Mitra N, Avril MF, Azizi E, Bergman W, Bianchi-Scarra G, Bishop DT, Bressac-de Paillerets B, Calista D, Cannon-Albright LA, Cuellar F, Cust AE, Demenais F, Elder DE, Friedman E, Gerdes AM, Ghiorzo P, Goldstein AM, Grazziotin TC, Hansson J, Hayward NK, Hocevar M, Hoiom V, Holland EA, Ingvar C, Landi MT, Landman G, Larre-Borges A, Leachman SA, Mann GJ, Nagore E, Olsson H, Palmer J, Peric B, Pjanova D, Puig S, Schmid H, van der Stoep N, Tucker MA, Wadt KAW, Whitaker L, Yang XR, Newton Bishop JA, Gruis NA, Kanetsky PA, Geno MELC (2016) Phenotypic and Histopathological Tumor Characteristics According to CDKN2A Mutation Status among Affected Members of Melanoma Families. J Invest Dermatol 136(5):1066-1069. 10.1016/j.jid.2016.01.009
19. Begg CB, Orlow I, Hummer AJ, Armstrong BK, Kricker A, Marrett LD, Millikan RC, Gruber SB, Anton-Culver H, Zanetti R, Gallagher RP, Dwyer T, Rebbeck TR, Mitra N, Busam K, From L, Berwick M, Genes E, Melanoma Study G (2005) Lifetime risk of melanoma in CDKN2A mutation carriers in a population-based sample. J Natl Cancer Inst 97(20):1507-15. 10.1093/jnci/dji312
20. Berwick M, Orlow I, Hummer AJ, Armstrong BK, Kricker A, Marrett LD, Millikan RC, Gruber SB, Anton-Culver H, Zanetti R, Gallagher RP, Dwyer T, Rebbeck TR, Kanetsky PA, Busam K, From L, Mujumdar U, Wilcox H, Begg CB (2006) The prevalence of CDKN2A germ-line mutations and relative risk for cutaneous malignant melanoma: an international population-based study. Cancer Epidemiol Biomarkers Prev 15(8):1520-5. 10.1158/1055-9965.Epi-06-0270
21. Orlow I, Begg CB, Cotignola J, Roy P, Hummer AJ, Clas BA, Mujumdar U, Canchola R, Armstrong BK, Kricker A, Marrett LD, Millikan RC, Gruber SB, Anton-Culver H, Zanetti R, Gallagher RP, Dwyer T, Rebbeck TR, Kanetsky PA, Wilcox H, Busam K, From L, Berwick M, Group GEMS (2007) CDKN2A germline mutations in individuals with cutaneous malignant melanoma. J Invest Dermatol 127(5):1234-43. 10.1038/sj.jid.5700689
22. FitzGerald MG, Harkin DP, Silva-Arrieta S, MacDonald DJ, Lucchina LC, Unsal H, O'Neill E, Koh J, Finkelstein DM, Isselbacher KJ, Sober AJ, Haber DA (1996) Prevalence of germ-line mutations in p16, p19ARF, and CDK4 in familial melanoma: analysis of a clinic-based population. Proc Natl Acad Sci U S A 93(16):8541-5. 10.1073/pnas.93.16.8541

23. McWilliams RR, Wieben ED, Rabe KG, Pedersen KS, Wu Y, Sicotte H, Petersen GM (2011) Prevalence of CDKN2A mutations in pancreatic cancer patients: implications for genetic counseling. Eur J Hum Genet 19(4):472-8. 10.1038/ejhg.2010.198
24. McWilliams RR, Wieben ED, Chaffee KG, Antwi SO, Raskin L, Olopade OI, Li D, Highsmith WE, Jr., Colon-Otero G, Khanna LG, Permuth JB, Olson JE, Frucht H, Genkinger J, Zheng W, Blot WJ, Wu L, Almada LL, Fernandez-Zapico ME, Sicotte H, Pedersen KS, Petersen GM (2018) CDKN2A Germline Rare Coding Variants and Risk of Pancreatic Cancer in Minority Populations. Cancer Epidemiol Biomarkers Prev 27(11):1364-1370. 10.1158/1055-9965.epi-17-1065
25. Stratigos AJ, Yang G, Dimisianos R, Nicolaou V, Stefanaki I, Katsambas AD, Tsao H (2006) Germline CDKN2A mutations among Greek patients with early-onset and multiple primary cutaneous melanoma. J Invest Dermatol 126(2):399-401. 10.1038/sj.jid.5700078
26. Aoude LG, Gartside M, Johansson P, Palmer JM, Symmons J, Martin NG, Montgomery GW, Hayward NK (2015) Prevalence of Germline BAP1, CDKN2A, and CDK4 Mutations in an Australian Population-Based Sample of Cutaneous Melanoma Cases. Twin Res Hum Genet 18(2):126-33. 10.1017/thg.2015.12
27. Burgstaller-Muehlbacher S, Marko M, Muller C, Wendt J, Pehamberger H, Okamoto I (2015) Novel CDKN2A mutations in Austrian melanoma patients. Melanoma Res 25(5):412-20. 10.1097/CMR.0000000000000179
28. Erlandson A, Appelqvist F, Wennberg AM, Holm J, Enerback C (2007) Novel CDKN2A mutations detected in western Swedish families with hereditary malignant melanoma. J Invest Dermatol 127(6):1465-7. 10.1038/sj.jid.5700718
29. Kannengiesser C, Dalle S, Leccia MT, Avril MF, Bonadona V, Chompret A, Lasset C, Leroux D, Thomas L, Lesueur F, Lenoir G, Sarasin A, Bressac-de Paillerets B (2007) New founder germline mutations of CDKN2A in melanoma-prone families and multiple primary melanoma development in a patient receiving levodopa treatment. Genes Chromosomes Cancer 46(8):751-60. 10.1002/gcc.20461
30. Kannengiesser C, Brookes S, del Arroyo AG, Pham D, Bombled J, Barrois M, Mauffret O, Avril MF, Chompret A, Lenoir GM, Sarasin A, French Hereditary Melanoma Study G, Peters G, Bressac-de Paillerets B (2009) Functional, structural, and genetic evaluation of 20 CDKN2A germ line mutations identified in melanoma-prone families or patients. Hum Mutat 30(4):564-74. 10.1002/humu.20845
31. Goldstein AM, Chan M, Harland M, Hayward NK, Demenais F, Bishop DT, Azizi E, Bergman W, Bianchi-Scarra G, Bruno W, Calista D, Albright LA, Chaudru V, Chompret A, Cuellar F, Elder DE, Ghiorzo P, Gillanders EM, Gruis NA, Hansson J, Hogg D, Holland EA, Kanetsky PA, Kefford RF, Landi MT, Lang J, Leachman SA, MacKie RM, Magnusson V, Mann GJ, Bishop JN, Palmer JM, Puig S, Puig-Butille JA, Stark M, Tsao H, Tucker MA, Whitaker L, Yakobson E (2007) Features associated with germline CDKN2A mutations: a GenoMEL study of melanoma-prone families from three continents. J Med Genet 44(2):99-106. 10.1136/jmg.2006.043802
